# Supplementary figures and images for: Prolonging culture of primary human keratinocytes isolated from suction blisters with the Rho kinase inhibitor Y-27632
Source: PLoS One. 2018 Sep 12;13(9):e0198862. doi: 10.1371/journal.pone.0198862 (PMC6135349; doi:10.1371/journal.pone.0198862)

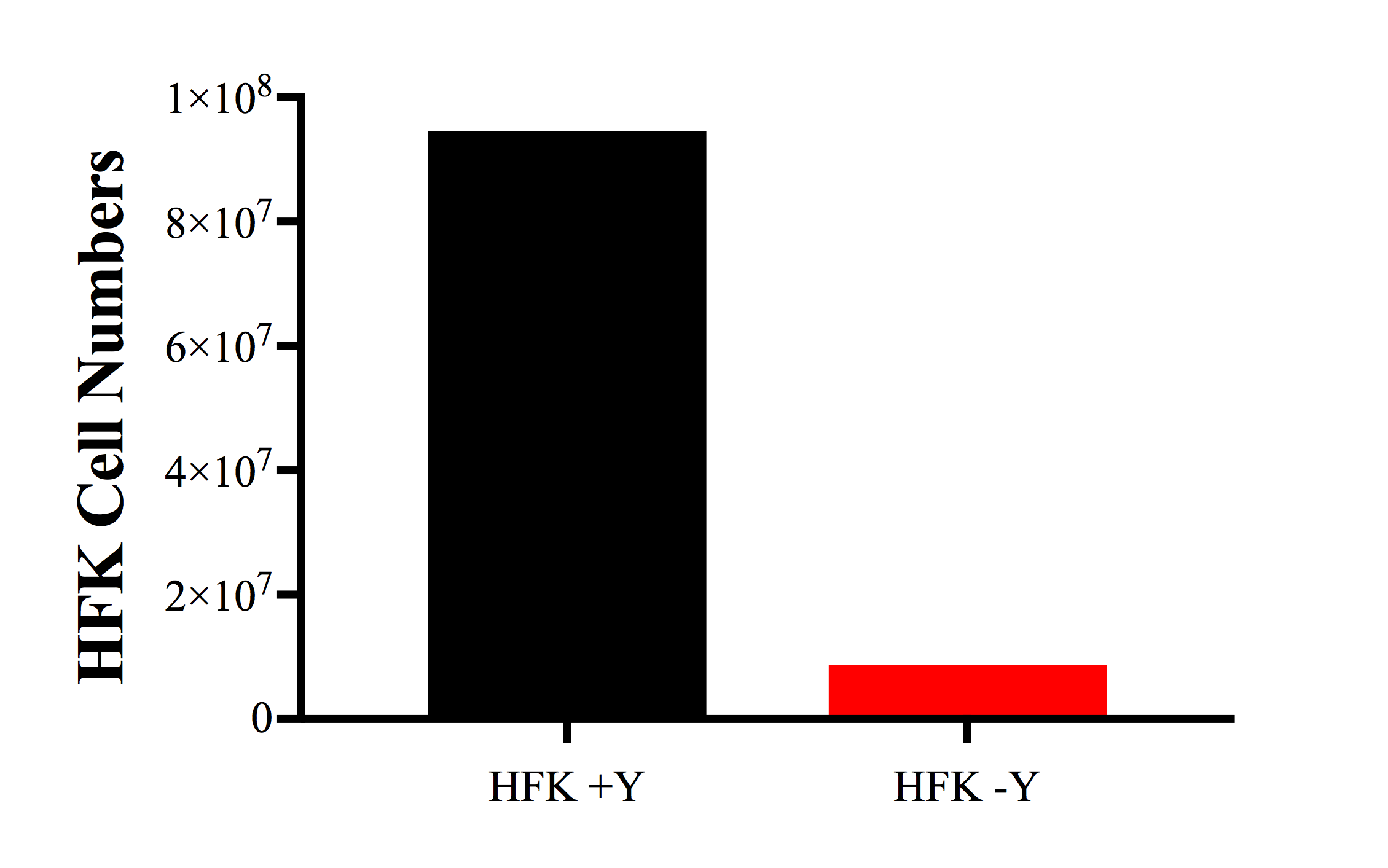

Supplement: S1 Fig — Sum total of HFK generated from the +Y (black) and -Y (red) conditions after 70 and 25 days respectively. (TIFF) [file pone.0198862.s001.tiff]

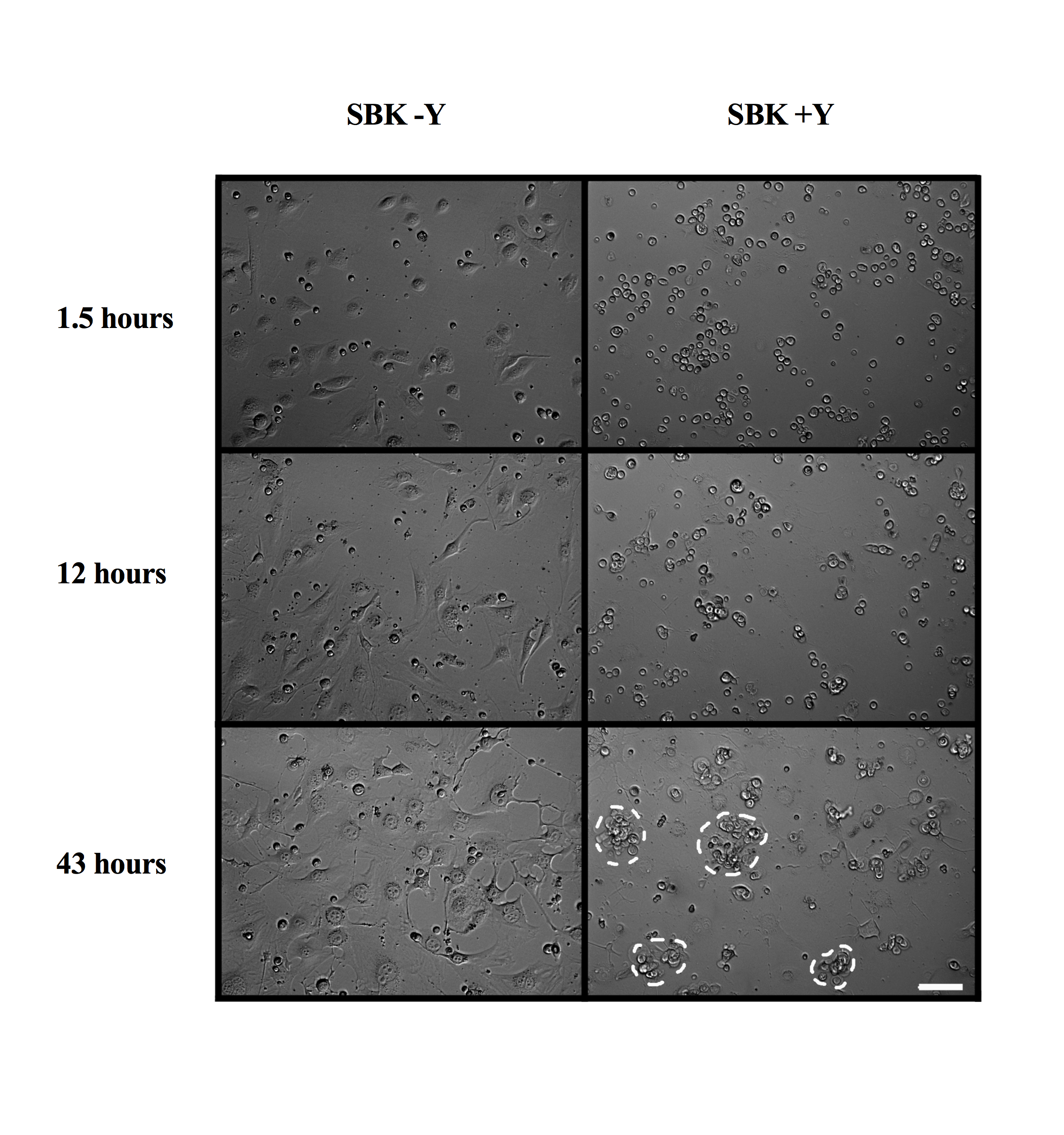

Supplement: S2 Fig — Time lapse of SBK cultured 1.5 hours after isolation from the patient, and with (+Y) or without (-Y) the Y-27632 inhibitor and monitored for colony formation. The dotted circles highlight colonies that are beginning to form after 43 hours in the +Y condition. (TIFF) [file pone.0198862.s002.tiff]

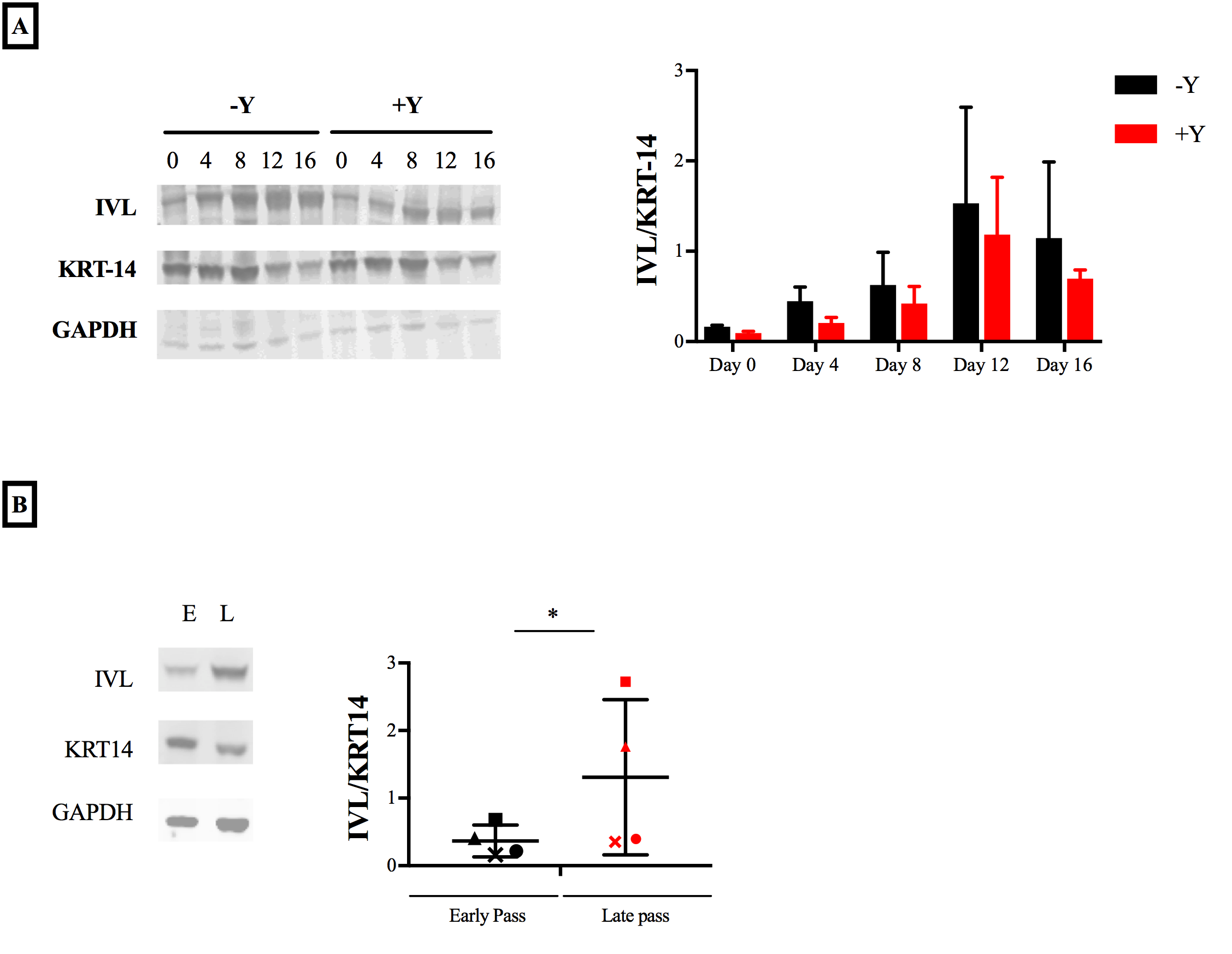

Supplement: S3 Fig — (A) KRT14 and IVL expression levels in SBK seeded in culture dishes and incubated with or without Y-27632 for 0, 4, 8, 12, or 16 days. The ratio of IVL/KRT14 ratios were calculated by integrated densitometry with ImageJ and normalized to GAPDH expression (right, n = 2 SBK patient cell lines were tested at passage 5). (B) KRT14 and IVL expression levels in early and late passage SBK cultured with Y-27632. Normalized IVL/KRT14 ratios are presented (right, n = 4,and each shape represents SBK from a different patient). *, p<0.05. (TIFF) [file pone.0198862.s003.tiff]
